# Evaluation of Sedentary Behavior and Physical Activity Levels Using Different Accelerometry Protocols in Children from the GENOBOX Study

**DOI:** 10.1186/s40798-021-00365-z

**Published:** 2021-11-24

**Authors:** Francisco Jesus Llorente-Cantarero, Jose Manuel Jurado-Castro, Rosaura Leis, Rocío Vázquez-Cobela, Esther M. González-Gil, Concepción María Aguilera, Gloria Bueno, Luis A. Moreno, Angel Gil, Mercedes Gil-Campos

**Affiliations:** 1 Department of Specific Didactics, Faculty of Education, University of Córdoba, Córdoba, Spain; 2 CIBEROBN, (Physiopathology of Obesity and Nutrition), Institute of Health Carlos III (ISCIII), Madrid, Spain; 3 Metabolism and Investigation Unit, Maimónides Institute of Biomedicine Research of Córdoba (IMIBIC), Reina Sofia University Hospital, University of Córdoba, Córdoba, Spain; 4 Pediatric Nutrition Research Group, Institute of Sanitary Research of Santiago de Compostela (IDIS), Unit of Pediatric Gastroenterology, Hepatology and Nutrition, Pediatric Service, Santiago de Compostela, University Clinical Hospital of Santiago (CHUS), Santiago, Spain; 5 GENUD Research Group, Instituto Agroalimentario de Aragón (IA2), Instituto de Investigación Sanitaria (IIS) Aragón, University of Zaragoza, Zaragoza, Spain; 6 Department of Biochemistry and Molecular Biology II, Center of Biomedical Research, Institute of Nutrition and Food Technology “José Mataix”, Instituto de Investigación Biosanitaria IBS, University of Granada, Granada, Spain; 7 Pediatric Endocrinology Unit, Lozano Blesa University Hospital, University of Zaragoza, Zaragoza, Spain

**Keywords:** Lifestyle, Activity monitor, Child, Exercise

## Abstract

**Background:**

Physical activity (PA) has acquired a significant relevance due to the health benefits associated with its practice. Accelerometers are an effective tool to assess PA; however, the diversity of cut-off points used to define different PA intensities through accelerometry could interfere in the interpretation of the findings among studies.

**Objectives:**

The present study aimed to examine the sedentary behavior (SB) and physical activity (PA) levels in children using six selected accelerometry protocols based on diverse cut-off points.

**Methods:**

Clinical examination, anthropometric measurements, and PA evaluation by accelerometry were assessed in 543 selected children (10 ± 2.4 years old) from the Spanish GENOBOX study. The ActiLife data scoring program was used to determine daily min spent in SB, and light, moderate, vigorous and moderate-vigorous PA using six validated accelerometry protocols differing in their cut-off points.

**Results:**

Very different estimations for SB and PA intensity levels were found in children, independently of the non-wear-time algorithm selected, and considering puberty stages, age and body mass index. The time spent in daily SB varied from 471 to 663.7 min, PA ranged from 141 to 301.6 min, and the moderate-vigorous PA was reported between 20.7 and 180.2 min.

**Conclusion:**

The choice of a particular accelerometry protocol considering these factors is important to evaluate SB or PA intensities to suit the characteristics of the sample researched. It seems necessary to establish future lines of research that include different analytical approaches to measure SB and PA by accelerometry based on standardized and validated methodology.

**Supplementary Information:**

The online version contains supplementary material available at 10.1186/s40798-021-00365-z.

## Key Points

- Estimated sedentary behavior and physical activity in children using six accelerometry protocols with different cut-off points, could lead to substantially different results. There were large differences by puberty stages, age and BMI category.
- The cut-off points proposed by Evenson et al. 2008 seems to be the most supported by the scientific community, due to the strict methodological and statistical procedures used in the validation of cut-off points.
- It seems necessary to establish future lines of research that include different analytical approaches to measure sedentary behavior and physical activity by accelerometry.

## Introduction

During the last decade, the term physical activity (PA) has acquired a significant relevance worldwide due to the health benefits associated with its practice [1–3]. The World Health Organization (WHO) recommends 60 min of moderate-vigorous physical activity (MVPA) per day in children [4]. However, a recent review revealed that globally, the practice of MVPA is below recommendations, being even lower in children with obesity (OB) than in their normal-weight (NW) peers [5].

In this way, valid, reliable and feasible measures are needed to quantify the actual prevalence of PA practice. Traditionally, the most used methods to measure PA in children have been self-reported PA questionnaires. Nevertheless, these methods present some limitations, such as their subjective character, the loss of information when children are not observed by their parents, such as the movement intensity or the real duration, or the difficulty of understanding the questions by scholars [6]. Consequently, researchers have looked for valid and reliable objective methods like accelerometry to assess PA in children despite this method has also some limitations [6, 7]. Accelerometers are medical-grade biometric monitoring devices that capture and record high-resolution raw acceleration data. These values are converted into objective activity and sleep measures using publicly available validated algorithms [8, 10]. Nowadays, these devices have a great quality of development, with a considerable number of data collection and processing capacity concerning specific criteria associated with SB and PA evaluations [9].

In relation to this, different cut-off points have been proposed in the literature to estimate SB and PA, therefore, there is no consensus on which cut-off points, accelerometer model, epoch length, non-wear-time (NWT) are the best to select [8–10]. This diversity of cut-off points used to define different PA intensities can interfere with interpreting the findings and comparing results between studies. This problem has been observed by different authors after the use of some accelerometry protocols in children.

Based on the results of previous studies [11, 12], the hypothesis of the present study is that differences in the estimation of SB and PA applying different accelerometry protocols could be relevant. There is still a need to refine the analytical approaches in the accelerometry methodology to better understand the influence of PA, especially on health outcomes in children [10]. The recent GRANADA consensus on accelerometry [10], determined to establish future lines of research that include different analytical approaches to measure SB and PA by accelerometry, such as average acceleration, scalar descriptors, MX metrics, as well as cut-off points. Therefore, the present work aimed to evaluate SB and PA levels in children of the Spanish GENOBOX study using only accelerometry protocols exclusively used for estimate PA in children and included in ActiLife have been selected, to evaluate if they presented differences classifying children by stage of puberty, age and BMI status.

## Materials and Methods

### Study Design and Study Population

The present study was carried out under the framework of the cross-sectional case–control GENOBOX study [13]. A subsample of children aged 6 to 14 years was selected for the present study using the following inclusion criteria: Children in good health and absence of endogenous OB, and having a minimal amount of useful accelerometer data of 8 h of monitoring per day for at least 3 days, including at least 1 weekend day. Exclusion criteria were disease or malnutrition and the use of medications that altered physiological or biochemical parameters.

The study was conducted in accordance with the Declaration of Helsinki. The Ethics Committees approved all experiments and procedures (Code IDs: Córdoba 01/2017, Santiago de Compostela 1011/198, Zaragoza 10/2010). All parents or guardians provided written informed consent, and the children gave their assent.

### Clinical Examination and Anthropometric Measurements

A medical history and a physical exam, including the evaluation of sexual maturity according to Tanner's five stages, were assessed [14]. Anthropometric measurements and blood pressure were taken by a single examiner. Details have been previously reported [13].

### Physical Activity Evaluation and Accelerometry Data Collection

PA was objectively evaluated using ActiGraph GT3 and GT3X+ accelerometers (ActiGraph; Pensacola, FL, USA). Raw data were collected by an ActiGraph device after it was assigned to a subject. The monitor measured accelerations in three individual axes (vertical, horizontal, perpendicular), with a dynamic range of ± 6 units of gravity, and was set to record at a frequency of 30 Hz. Parents and children were instructed to wear the ActiGraph 24 h per day, 7 days, on the iliac crest on the right hip with an elastic belt. The device could be removed only during shower or nocturnal rest (if the instrument caused discomfort during sleep).

#### Accelerometry Data Processing and Cut-Off Points Selection

Summary of 'epoch' data are essentially raw data that have been filtered in order to run it through algorithms to produce outputs. An epoch is a date and time from which a computer measures time; in practice, it is the number of seconds that have elapsed between two consecutive measurements. The raw data are summed into chunks of data ('epochs'), and the *G* values (universal gravitational constant to determine the intensity of the gravitational acceleration force) are converted to activity counts. Summary data are used to generate the results shown in ActiLife. ActiLife version 6.13.3 (ActiGraph Software Department: Pensacola, FL, USA) was used to download these data from the monitor at 15 epochs.

#### Non-wear-Time Validation

The non-wear-time (NWT) validation tool in ActiLife allows users to easily flag invalid data (or data collected when a device was unworn) for exclusion from further analysis. The NWT was defined as an interval of consecutive min (min) of zero activity intensity counts. ActiLife offers the possibility to schedule the Data Screening Criteria or establish default options to determine NWT. As default, the program provides two possibilities: (a) Troiano 2007 criteria [15] included a minimum length of 60 min, 2 min of spike tolerance [16], 100 counts per min of spike level to stop and require consecutive epochs outside the activity threshold (this criterion is cataloged as NWT-60 from now onwards); (b) Choi et al. 2011 criteria [17] included a minimum length of 90 min (NWT-90), a small window length of 30 and 2 min of spike tolerance. Trying to select one of them, a random selected subsample was analyzed using those authors' criteria (NWT-60 vs. NWT-90) obtaining very similar results. Following with the Data Screening Criteria, a growing trend recommends establishing as criteria a minimum length of 20 min and 0 min for the rest of the options in children (this criterion is cataloged as NWT-20 from now onwards) [7]. ActiLife allows users to customize the non-wear periods, but only under Troiano and Choi’s algorithms [15, 17]. Hence, the random subsample was analyzed again by both algorithms but scheduled by the NWT-20 criteria. Selecting the option by Choi et al. [17], the software did not distinguish between wear and no-wear, understanding all-time analyzed as wear period. Therefore, it was decided to include in the final analysis only Troiano’s algorithms by NWT-20 [7] and NWT-60 [15]. In addition to the previously described criteria, Vector Magnitude and the following Optional Screen Parameters were selected: ignoring wear periods less than 20 min and sleep periods; performing a minimum of 8 h of monitoring per day for at least 3 days, including at least 1 weekend day [18]. These periods were replaced as missing data codes before downstream analysis.

#### Scoring

Traditionally, SB and PA have been estimated based on the number of CPM accumulated in a given period (length of time). The cut-off points are the thresholds of the activity counts used to categorize the activity as LPA, MPA and VPA.

Exclusion criteria for this analysis included unavailability of valid data, non-compliance with the minimum number of hours set or if there was not enough time on valid days during the week or weekend, described above. All cut-off point values in ActiLife are based on 60-s epoch lengths. When sub-60-s files are used, ActiLife scales the epoch count level up to its 60-s equivalent before performing the cut point categorization. Files using epochs larger than 60-s have not been calculated. Also, to exclude low-quality records, all negative counts were replaced by missing data code.

The ActiLife data scoring program was used to determine daily minspent in SB, LPA, MPA, VPA, MVPA for each epoch length dataset, using the Evenson et al. [18], Pulsford et al. [19], Freedson et al. [20], Puyau et al. [21], Mattocks et al. [22], Troiano et al. [15] activity cut-off points accelerometry protocols that were defined as different intervals of counts per minute (CPM). The accelerometry protocols considered were those included in ActiLife for the PA estimation in children and moreover providing cut-off points for the different PA intensities for this age range. So, other protocols without these criteria were excluded. For those accelerometry protocols with criteria established in 15-s, it was necessary to transform to 4 periods of 15 epoch in one period of 60 epoch. These protocols were validated with accelerometers of a vertical axis (Table 1).

**Table 1:** Characteristics for different accelerometry protocols and data for physical activity intensity levels in children from the Genobox study according to them

Accelerometry protocol	Population (age)	Sample (*n*)	Placement	Epoch (s)^‡^	Device used	Sedentary CPM	Light PA CPM	Moderate PA CPM	Vigorous PA CPM	Very-vigorous PA CPM
Evenson et al. [18]	5–8 years	33	Right hip	15*	ActiGraph MT1 7164	≤ 100	101–2295	2296–4011	≥ 4012	NE
Freedson et al. [20]	6–18 years	538^^^	Right hip	60	ActiGraph /Actical/Actiwatch/RT3 Triaxial	≤ 149	150–499	500–3999	4000–7599	≥ 7600
Mattocks et al. [22]	12 years	83	Right hip	60	ActiGraph MT1 7164	≤ 100	101–3580	3581–6129	≥ 6130	NR
Pulsford et al. [19]	7–8 years	53	Right hip	15*	Actigraph GT1M 1X	≤ 100	101–2240	2241–3840	≥ 3841	NR
Puyau et al. [21]	6–16 years	26	Right hip	60	ActiGraph MT1 7164	≤ 799	800–3199	3200–8200	≥ 8200	NR
Troiano et al. [15]	6–11 years/12–19/20+	6329^**†**^	Right hip	60	ActiGraph MT1 7164	≤ 99	100–2019	2020–5998	≥ 5999	NR

*CPM* counts per min, *NE* non-established category by original authors, *PA* physical activity

^^^The sample size was composed of the sum of different studies selected to establish the validation protocol

^†^Data are described from 6329 participants who provided at least 1 d of accelerometer data and from 4867 participants who provided four or more days of accelerometer data

^‡^Number of seconds that have elapsed between two consecutive measurements

*These units were initially displayed in Epoch 15 s and transformed to Epoch 60 s in ActiLife

### Statistical Analysis

The sample size estimation was calculated for the GENOBOX study as reported elsewhere [13]. All continuous variables were tested for normality using the Shapiro–Wilk test. Heteroskedasticity between experimental groups was explored with the Levene test. Two-way ANOVA and Wilcoxon tests, depending on variables following or not a normal distribution, with repeated measures were applied to compare mean SB and PA intensities among the different accelerometry protocols.

One-way ANOVA and the Kruskal–Wallis tests, depending on variables following or not a normal distribution, were employed to assess differences in SB and PA levels between OB, OW and NW, as well as prepubertal and pubertal stages and age quartiles. Pairwise analysis adjusted by BMI *Z*-score and age were applied conveniently as post hoc analyses to determine which experimental groups differed from each other. Values in descriptive tables and results are expressed as means and standard deviations. A *p* value < 0.05 was considered significant.

Additionally, Bland–Altman plots were created to assess the level of agreement between the accelerometry protocol of Evenson et al. [18] compared with the others. Evenson et al. [18] was selected as a reference method for performing the Bland–Altman test, due to the strict methodological and statistical procedures used in the validation of cut-off points. The one-sample *t* test was used to determine whether there were statistically significant differences between the mean of the scanning accelerometry protocols. All statistical procedures were conducted by using SPSS (IBM SPSS Statistics, Version 25.0. Armonk, NY, USA).

## Results

### Demographic and Anthropometric Data

A description of the 543 participants' characteristics is shown in Table 2. Two hundred seventy-four participants were prepubertal (50.4%). Within the sample, 313 were OB (57.5%), 109 OW (20%) and 121 NW (22.5%).

**Table 2:** Descriptive characteristics of the selected children within the GENOBOX study

Variable	Females	Males
*n*	286	257
Age (years)	10.6 ± 2.4	10.8 ± 2.4
Height (m)	1.44 ± 0.14	1.46 ± 0.15
Weight (kg)	54.7 ± 19.4	54.7 ± 21
BMI (kg/m^2^)	25.01 ± 5.6	24.6 ± 5.8
BMI *Z*-Score (index)	2.1 ± 2.2	1.9 ± 1.9
Hip circumference (cm)	92.9 ± 9.2	89.6 ± 14.1
Waist circumference (cm)	82.4 ± 15.3	82.6 ± 16.1
Waist-to-height ratio (index)	0.57 ± 0.09	0.56 ± 0.09
Systolic blood pressure (mmHg)	109.7 ± 12	110.5 ± 14.9
Diastolic blood pressure (mmHg)	65.8 ± 9.4	65.9 ± 10.4

Data expressed as mean ± standard deviation

*BMI* body mass index

### Non-wear-Time, Sedentary Behavior and Physical Activity Levels by Accelerometry Protocols

The accelerometer wearing time was 4.8 ± 0.8 days. The number of participants meeting the NWT criteria was very similar in both NWT-20 and NWT-60 (535 vs. 543, respectively). Moreover, on average, both criteria accumulated the same number of days with valid data. The accelerometry protocols showed differences (*p* < 0.05) when comparing the results obtained under NWT-20 versus NWT-60 criteria (Table 3). Regardless of NWT criteria, higher SB were obtained for Puyau et al. [21], higher light PA (LPA) for Mattocks et al. [22], and higher moderate PA (MPA) and MVPA for Freedson et al. [20] (Table 3).

**Table 3:** Minutes/day estimated in sedentary behavior and physical activity intensities by different accelerometry protocols in children of the GENOBOX study

Accelerometry protocol	Sedentary behavior (min)	Light PA (min)	Moderate PA (min)	Vigorous PA (min)	Moderate–vigorous PA (min)
Removed periods of 20 min or more of consecutive zero counts (NWT-20) (*n* = 535)					
Evenson et al. [18]	473.4 ± 89.9^a^	247.6 ± 61.2^a^	36.4 ± 13.9^a^	15.3 ± 21.3^a^	51.6 ± 27.7^a^
Freedson et al. [20]	498.1 ± 88.7^c^	92.5 ± 22.5^b^	166.5 ± 46.6^b^	13.6 ± 10.1^b^	180.2 ± 50.3^b^
Mattocks et al. [22]	473.4 ± 89.9^a^	278.2 ± 67.5^c^	17.6 ± 11.4^c^	3.4 ± 18.4^c^	21 ± 22.5^c^
Pulsford et al. [19]	471 ± 90^b^	248.2 ± 61.2^d^	36.1 ± 13.5^d^	17.3 ± 21.7^d^	53.4 ± 28^d^
Puyau et al. [21]	631.5 ± 82.8^d^	113.2 ± 34.2^e^	26.3 ± 15.2^e^	1.5 ± 17.4^e^	27.8 ± 23.7^e^
Troiano et al. [15]	471 ± 90^b^	239.8 ± 59.1^f^	58.1 ± 22.6^f^	3.7 ± 18.5^f^	61.8 ± 29.6^f^
*p* value*	< 0.001	< 0.001	< 0.001	< 0.001	< 0.001
Removed periods of 60 min or more of consecutive zero counts (NWT-60) (*n* = 543)					
Evenson et al. [18]	507.1 ± 117.6^a^	245.2 ± 61.1^a^	36 ± 13.7^a^	15 ± 21.1^a^	51 ± 27.5^a^
Freedson et al. [20]	531.6 ± 116.5^c^	91.6 ± 22.4^b^	165 ± 46.6^b^	13.4 ± 10^b^	178.4 ± 50.3^b^
Mattocks et al. [22]	507.1 ± 117.6^a^	275.5 ± 67.5^c^	17.4 ± 11.3^c^	3.4 ± 18.3^c^	20.7 ± 22.3^c^
Pulsford et al. [19]	504.7 ± 117.7^b^	245.8 ± 61.1^d^	35.7 ± 13.3^d^	17.1 ± 21.6^d^	52.8 ± 27.8^d^
Puyau et al. [21]	663.7 ± 110.4^d^	112.2 ± 34.1^e^	25.9 ± 15.1^e^	1.5 ± 17.3^e^	27.4 ± 23.6^e^
Troiano et al. [15]	504.7 ± 117.7^b^	237.5 ± 59^f^	57.5 ± 22.4^f^	3.6 ± 18.3^f^	61.1 ± 29.4^f^
*p* value*	< 0.001	< 0.001	< 0.001	< 0.001	< 0.001

Data expressed as mean ± standard deviation

*PA* physical activity

No matching superscript letters (a, b, c, d, e, f) indicate significant differences in sedentary behavior or different intensities of physical activity between the different accelerometry protocols by two-way ANOVA and Wilcoxon tests, depending on variables following or not a normal distribution, with repeated measures (*p* < 0.05)

*p value between the accelerometer protocols for sedentary behavior and different physical activity intensity levels applying a one-way ANOVA

Comparing the accelerometry protocols among them, independently of NWT criteria, differences were observed for SB variables as well as for PA intensities except Evenson et al. [18] versus Mattocks et al. [22]; and Pulsford et al. [19] versus Troiano et al. [15] (only for SB) (Table 3). These differences between accelerometry protocols were reproduced when comparing by pubertal stage, age or BMI classification (results not shown).

The one-sample *t* test results (reference = 0) showed significant differences on SB and different PA intensities between Evenson et al. [18] compared with the others accelerometry protocols, with exception on SB for Evenson et al. [18] versus Mattocks et al*.* [22]. These differences revealed by Bland–Altman plots are shown as Additional file 1.

### Sedentary Behavior and Physical Activity Levels According to Puberty Stage

**Fig. 1:**
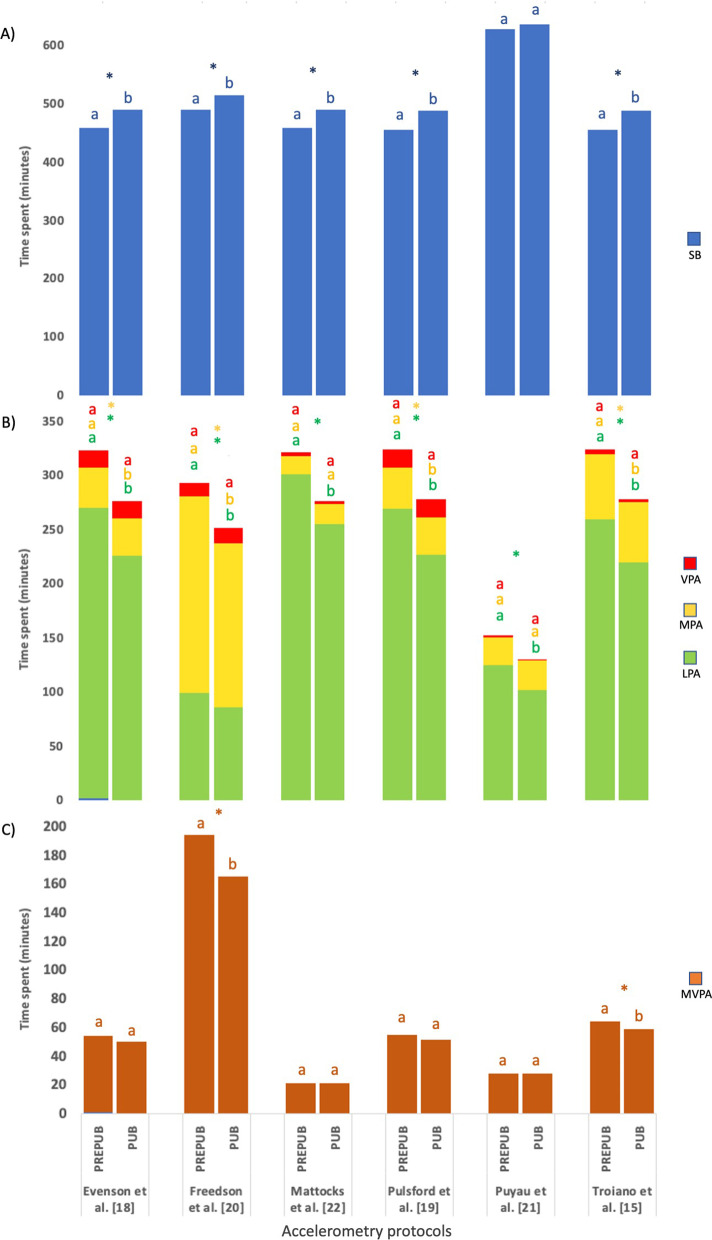
Min/day spent in sedentary behavior and physical activity in prepubertal and pubertal children of the GENOBOX study, estimated using selected accelerometry protocols. **A** Min/day spent in sedentary behavior; **B** min/day spent in light, moderate and vigorous physical activity; **C** min/day spent in moderate-vigorous physical activity. *LPA* light physical activity, *MPA* moderate physical activity, *MVPA* moderate-vigorous physical activity, *PREPUB* prepubertal, *PUB* pubertal, *SB* sedentary behavior, *VPA* vigorous physical activity. *Differences (*p* < 0.05) were determined by one-sample *t* test between prepubertal and pubertal stages applying 20 min non-wear-time criteria. No matching superscript letters (a, b) indicate significant differences by pairwise applying 20 min non-wear-time. *Note*: These shown data had the same behavior for the 60-min non-wear-time (results not shown)

Prepubertal children, regardless of NWT criteria, had lower SB (Fig. 1A) and higher LPA, MPA (Fig. 1B) and MVPA (Fig. 1C), in most accelerometry protocols, than pubertal children (*p* < 0.05).

### Sedentary Behavior and Physical Activity Levels According to Age

**Fig. 2:**
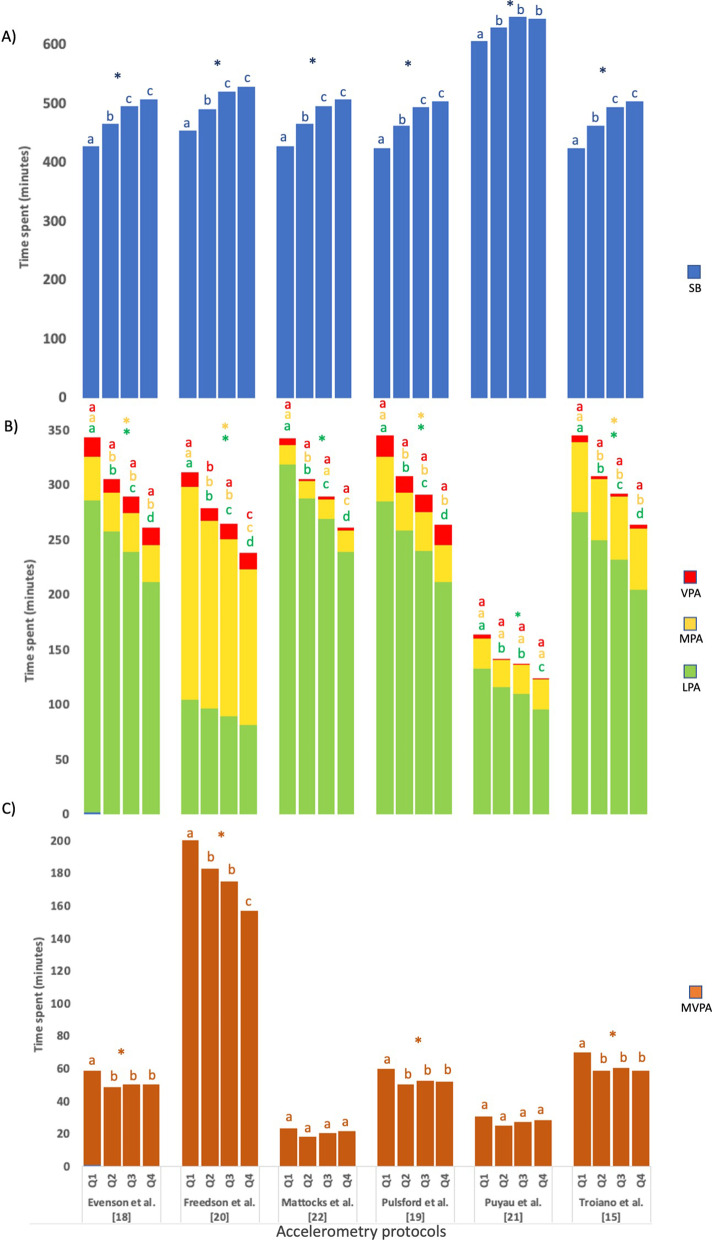
Min/day spent in sedentary behavior and physical activity among quartiles of age in children of the GENOBOX study, estimated using selected accelerometry protocols. **A** Min/day spent in sedentary behavior; **B** min/day spent in light, moderate and vigorous physical activity; **C** min/day spent in moderate-vigorous physical activity. *PA* light physical activity; *Q*1: < 10 year-old; *Q*2: 10–11.7-year-old; *Q*3: 11.7–13.7-year-old; *Q*4: 13.7-year-old; *MPA* moderate physical activity, *MVPA* moderate-vigorous physical activity; *SB* sedentary behavior, *VPA* vigorous physical activity. *Age quartiles differences (*p* < 0.05) applying 20 min non-wear-time criteria by a one-way ANOVA. No matching superscript letters (a, b, c, d) indicate significant differences by pairwise applying 20 min non-wear-time. *Note*: Data presented in the figure had the same behavior that those obtained with the 60-min non-wear-time (results not shown)

The sample was divided into age ranges and classified by quartiles (*Q*): *Q*1 (< 10 years); *Q*2 (10–11.7 years); *Q*3 (11.7–13.7 years); *Q*4 (> 13.7 years). The sample size in these quartiles was as follows: *Q*1, *n* = 136; *Q*2, *n* = 131: *Q*3, *n* = 140; *Q*4, *n* = 136. Regardless of NWT-20 and NWT-60, more SB (*p* < 0.05) (Fig. 2A) and lower LPA and MPA (*p* < 0.05) were observed as the age quartile increased (from *Q*1 to *Q*4). However, no age-related differences were seen for vigorous PA (VPA) (Fig. 2B) (*p* < 0.05). Children in *Q*1 showed higher MVPA (*p* < 0.05) in the accelerometry protocols of Evenson et al. [18], Freedson et al. [20], Pulsford et al. [19] and Troiano et al. [15] (Fig. 2C).

### Sedentary Behavior and Physical Activity Levels According to BMI Category

**Fig. 3:**
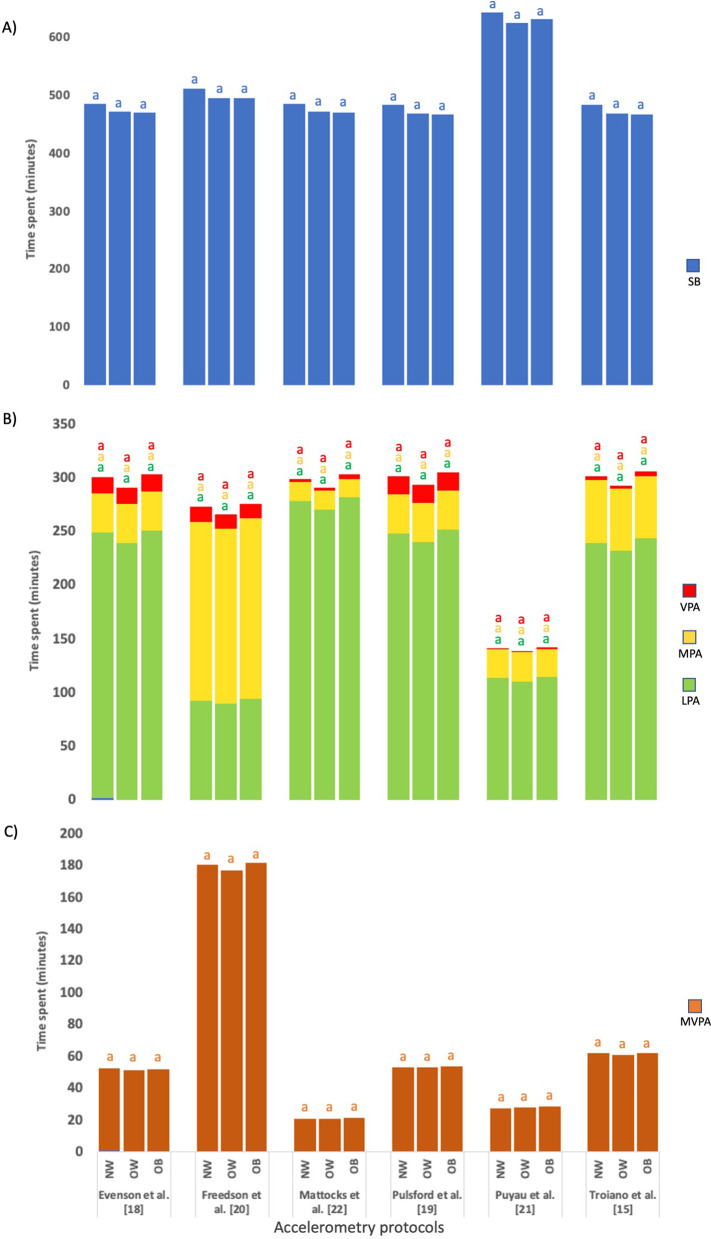
Min/day spent in sedentary behavior and physical activity between BMI category in children of the GENOBOX study classified on BMI, estimated using selected accelerometry protocols. **A** Min/day spent in sedentary behavior; **B** min/day spent in light, moderate and vigorous physical activity; **C** min/day spent in moderate-vigorous physical activity. *LPA* light physical activity, *MPA* moderate physical activity, *MVPA* moderate-vigorous physical activity, *SB* sedentary behavior, *VPA* vigorous physical activity. No matching superscript letters would indicate significant differences by pairwise applying 20 min non-wear-time. There are not significant differences. *Note*: Data in this figure had the same behavior as that obtained with the 60-min of non-wear-time (results not shown)

Neither other differences were observed when comparing the BMI category, regardless of NWT, in SB min, nor PA intensities within each accelerometry protocol (Fig. 3A–C).

## Discussion

The recent GRANADA consensus on accelerometry [9] was addressed to establish future lines of research that include different analytical approaches, as well as cut-off points, to measure SB and PA by accelerometry. Therefore, the present study compares the application of six validated accelerometry protocols based on specific cut-off points to evaluate PA, showing very different estimations for SB and PA intensity levels in children; even considering the epoch lengths used in their validation studies; and independently of the NWT algorithm selected, NWT-20 or NWT-60, or the puberty stage, age and BMI.

One of the first aspects to be considered is the selection of the two possible criteria for NWT, the NWT-20 proposed by Cain et al. [7] or the NWT-60 proposed by Troiano et al. [15]. Both proposals showed several differences when compared among authors, with the exception that NWT-60 criteria led to a higher SB, and NWT-20 criteria that accumulated more time on PA intensities. For adults, NWT-20 has shown the lowest misclassification error, although it presents the inconvenience that it may result in slightly greater data loss (6% of the sample size) [10, 23]. As the precision between NWT-20 and NWT-60 seems similar, the literature has suggested using NWT-60 without allowing interruptions in the collect criterion of counts as a general recommendation for adults [10]. However, in children, it could be very different. Thus, in our study, only a loss of 1.5% of the participants (*n* = 8) with NWT-20 was detected. Therefore, it seems more adequate NWT-20 for children. However, more studies are needed to examine the accuracy of different NWT detection algorithms in all age groups of children and adolescents.

Although it is difficult to establish a recommendation, in the present study, there are differences between both NWT. These results might be due to the time interval that must elapse without counts for NWT-60 is greater than in NWT-20; so, NWT-60 criteria may be interpreting this time as SB instead of NWT. Although children or adolescents with OW or OB might present a longer time of consecutive 0 counts per minute (CPM) associated with a higher SB [24], especially in prepubertal children [25], this time could be misclassified as NWT.

Traditionally, SB and PA intensity have been estimated based on the number of CPM accumulated in a given period (length of time). The cut-off points are the thresholds of the activity counts used to categorize the activity as light, moderate and vigorous PA. This study selected 6 validated protocols based on different cut-off points and standards for PA interpretation. Although other standard measures can be found in the literature with similar mean cut-off points [7, 10]_,_ the accelerometry protocol criteria were selected to represent the group of protocols more frequently used to estimate PA in school-age children, mainly with Actigraph accelerometers. These are also the protocols provided by ActiLife for estimating PA in school-aged children [7, 10].

To calibrate the different range of accelerometer counts corresponding to predefined SB, the intensity levels or to estimate energy expenditure, authors usually involved movements as walking, running or stationary bicycle (only in the case of Evenson et al. [18]) alone or in combination with free-living activities (TV watching, arts and crafts) [21] in their study protocols. However, the methods used to analyze and quantify the physiological response of participants were different in each accelerometry protocol, such as: oxygen consumption (VO2) and the heart rate [18]; refitting the energy expenditure model with VO_2_ as the outcome [20]; calibrated against energy expenditure measures (kcal kg^−1^ h^−1^) obtained over a range of exercise intensities using a COSMED K4b2 portable metabolic unit [19]; 6-h energy expenditure measurements by room respiration calorimetry, activity by microwave detector, and heart rate by telemetry [22]; reviewed the calibration of different accelerometers used most frequently to assess PA and SB in children [21]; or based on the results of the National Health and Nutritional Examination Survey (NHANES)’s [15]. Although most of the accelerometry protocols used objective, validated, and standardized methods to associate the movement with their physiological response, Puyau et al. [21] seems to present a more controlled environment, specially to measure the SB, furthermore, Evenson et al. [18] used a robust statistical analysis compared to other accelerometry protocols.

On the other hand, the accelerometry protocols included in the present study only used the vertical axis to measure the movement in the validation of their protocols. Nevertheless, the current Actigraph models (as GT3X) also include two more axes. Even though it has been verified that the Actigraphs with a single vertical axis are comparable with those with a triaxial axis [26, 27], new protocols are trying to get recognition by the scientific community and the Actigraph Corporation for the GT3X model [28–31]. These accelerometry protocols were not considered for the present study, as they did not provide cut-off points for the different PA intensities.

The number of epochs established at the set-up also seems to determine the protocol precision. In a recent systematic review, Migueles et al. [10] recommend for children the Hänggi et al. [32] cut-off points developed in 1-s epoch for the hip due to the excellent classification accuracy (ROC-AUC > 0.90 for all cut-points) obtained and the cover for almost the whole spectrum of PA intensities. The ranges obtained by Hänggi et al. [32] were “< 3 counts for SB, 3–56 counts for LPA and > 56 counts for MVPA”. If values from 1-s epoch to 60-s epochs are transformed, the results are within the following ranges “< 180 for SB, 180–3360 for LPA and > 3360 for MVPA”. These are very similar to those proposed by Mattocks' et al. [22] (SB: ≤ 100; LPA: 101–3580; MPA: 3581–6129; VPA: ≥ 6130), that was the protocol included in the present study. The latter has the advantage of being able to study separately MPA and VPA intensities.

Once analyzed the accelerometer protocols, it seems that the protocols are roughly comparable with the exception of that Puyau et al. [21] which overestimated SB compared to the others, and Freedson et al. [20] that overestimated MPA, and consequently MVPA. In this sense, it was found that the lower value in min obtained for SB was 471/504.7 min [19] versus 631.5/663.7 min in the upper value [21], respectively, for NWT-20 and NWT-60, highlighting the MVPA which was 21/20.7 min [22] versus 180.2/178.4 min [20]. Moreover, Freedson et al. [20] and Puyau et al. [21] underestimated LPA, and Mattocks et al. [22] underestimated VPA (Table 3). These results were a direct result of the different cut-off points published between the different protocols (Table 1). Indeed, the current literature reporting PA children data measured by accelerometry must be interpreted with caution, paying attention to the analysis protocol when comparing one study's results with others [11].

To evaluate PA in children, it is essential to consider age and puberty stage. All selected accelerometry protocols for this study included criteria for school-age children and some of them for adolescents. Although the puberty stages were not specified in the protocols' validation, the age range was between 5 and 19 years. Usually, the studies include an age range higher than a couple of years and usually comprises children from 5–6 to 14. During puberty, males gain greater amounts of fat free mass and skeletal mass, whereas females acquire significantly more fat mass [33]. Therefore, an age range very wide, e.g., 6–18 years, may lead to a less specific measure. Only Mattocks et al. [22], and Puyau et al. [21] did not show differences when compared by puberty stage or age quartiles in the MPA and MVPA intensities, for both NWT-20 and NWT-60. This may indicate that both accelerometry protocols seem to be less precise for ages outside those included in their protocols.

Other consideration is that all the selected protocols used children with NW to validate their cut-off, except for Troiano et al. [15], who included 2–3% of OW, but no OB. Despite establishing various cut-off by each accelerometry protocol, no differences were obtained by BMI category in SB and the different PA intensities comparing them. The fact that none of the accelerometry protocols has included OB children in their validation raises the question whether the estimates of SB and PA in children with OB measured with accelerometry are reliable or not, considering that this methodology is commonly used in the evaluation or in interventions related to childhood obesity [34]. This question has not been exactly resolved so far, although few studies have provided approximations and interesting data [12, 35–37]. Robertson et al. [35] conducted an investigation only in children with OB, concluding that accelerometers are acceptable to most of the children, although their use at school is problematic for some of them because they may underestimate children's PA, as some children with OB are unwilling to wear accelerometers at school and during sports because they feel they are at risk of stigma and bullying. The aim of Moura et al. study [36] was to analyze the impact of cut-off points in defining SB time and prevalence in adolescents from Northeastern Brazil. Also in this context, Migueles et al. [12] aimed to examine how cut‐points relative to different attachment sites affect the final estimations of SB and PA in children with overweight/OB. Similar to our study, the cut-off points examined by them produced significant differences in SB and PA estimates. Gaba et al. [37] reported a curvilinear analysis that indicated the optimal thresholds for CPM and MVPA derived from the Puyau et al. [21], which was very useful in classifying children according to their BMI and fat mass percentage to overweight and obesity prevention but only considering MVPA.

According to the Bland–Altman plots, the accelerometry protocol of Evenson et al. [18] method showed large mean differences with that of Puyau et al. [21] for SB and LPA, which had a more controlled environment to validate cut-off points with energy expenditure. However, despite there being also differences, the mean differences were lower for Evenson et al. [18] versus Puyau et al. [21], and Evenson et al. [18] versus Mattocks et al. [22] for MPA, VPA and MVPA.

As a limitation of our study, only the accelerometry protocols available in ActiLife were included, validated with a vertical axis. Other validated protocols with triaxial axis were not included due to the lack of cut-off points for all PA intensities. Future research should focus on validating cut-off points for all PA intensities considering also triaxial axis and assessing new analysis metrics for estimating PA in children, such as average acceleration, scalar descriptor or MX metrics [9].

## Conclusions

In conclusion, although cut-off points proposed by Evenson et al. [18] seem to be the most supported by the present study and the scientific community, the high differences between the accelerometry protocols to evaluate physical activity, currently used represent an important gap in this scientific field. The present study finds that data processing and analysis of sedentary behavior and physical activity intensity levels in children using six accelerometry protocols could lead to substantially different results, especially when puberty, age and BMI category are considered. It seems that the protocols are roughly comparable with the exception that Puyau et al. [21] which seems to overestimate sedentary behavior, and Freedson et al. [20] which seems to overestimate moderate or high intensities. However, it seems necessary to establish future lines of research that include different analytical approaches to evaluate physical activity by accelerometry such as average acceleration, scalar descriptors, MX metrics, as well as cut-off points.

## Supplementary Information

**Additional file 1. Supplementary Figure 1**. Bland-Altman plots of sedentary behavior measurement agreement between the Evenson et al. and Freedson et al.; Mattocks et al.: Pulsford et al.; Puyau et al.; Troiano et al. accelerometry protocols. SB; sedentary behavior.** Supplementary Figure 2**. Bland-Altman plots of light activity measurement agreement between the Evenson et al. and Freedson et al., Mattocks et al., Pulsford et al., Puyau et al., Troiano et al. accelerometry protocols. LPA: light physical activity.** Supplementary Figure 3**. Bland-Altman plots of moderate physical activity measurement agreement between the Evenson et al. and Freedson et al., Mattocks et al., Pulsford et al., Puyau et al., Troiano et al. accelerometry protocols. MPA: moderate physical activity.** Supplementary Figure 4**. Bland-Altman plots of vigorous physical activity measurement agreement between the Evenson et al. and Freedson et al., Mattocks et al., Pulsford et al., Puyau et al., Troiano et al. accelerometry protocols. VPA: vigorous physical activity.** Supplementary Figure 5**. Bland-Altman plots of moderate-vigorous physical activity measurement agreement between the Evenson et al. and Freedson et al., Mattocks et al., Pulsford et al., Puyau et al., Troiano et al. accelerometry protocols. MVPA: moderate-vigorous physical activity.

## Data Availability

Not applicable.

## Abbreviations

BMI: Body mass index
CPM: Counts per minute
LPA: Light physical activity
MPA: Moderate physical activity
MVPA: Moderate-vigorous physical activity
NWT: Non-wear-time
NWT-20: 20 min of non-wear-time
NWT-60: 60 min of non-wear-time
NW: Normoweight
PA: Physical activity
SB: Sedentary behavior
OB: Obesity
OW: Overweight
VPA: Vigorous physical activity

## References

[1] Westerterp KR (2013). Physical activity and physical activity induced energy expenditure in humans: measurement, determinants, and effects. Front Physiol.

[2] Reiner M, Niermann C, Jekauc D (2013). Long-term health benefits of physical activity—a systematic review of longitudinal studies. BMC Public Health.

[3] Warburton DER, Bredin SSD (2017). Health benefits of physical activity. Curr Opin Cardiol.

[4] Bull FC, Al-Ansari SS, Biddle S (2020). World Health Organization 2020 guidelines on physical activity and sedentary behaviour. Br J Sports Med.

[5] Elmesmari R, Martin A, Reilly JJ (2018). Comparison of accelerometer measured levels of physical activity and sedentary time between obese and non-obese children and adolescents: a systematic review. BMC Pediatr.

[6] Jurado-Castro JM, Llorente-Cantarero FJ, Gil-Campos M (2019). Evaluación de la actividad física en niños. Act Pediat Esp.

[7] Cain KL, Sallis JF, Conway TL (2013). Using accelerometers in youth physical activity studies: a review of methods. J Phys Act Health.

[8] Galland BC, Kennedy GJ, Mitchell EA (2012). Algorithms for using an activity-based accelerometer for identification of infant sleep–wake states during nap studies. Sleep Med.

[9] Migueles JH, Aadland E, Andersen LB (2021). GRANADA consensus on analytical approaches to assess associations with accelerometer-determined physical behaviours (physical activity, sedentary behaviour and sleep) in epidemiological studies. Br J Sports Med.

[10] Migueles JH, Cadenas-Sanchez C, Ekelund U (2017). Accelerometer data collection and processing criteria to assess physical activity and other outcomes: a systematic review and practical considerations. Sports Med.

[11] Mota J, Valente M, Aires L (2007). Accelerometer cut-points and youth physical activity prevalence. Eur Phys Educ Rev.

[12] Migueles JH, Cadenas-Sanchez C, Tudor-Locke C (2018). Comparability of published cut-points for the assessment of physical activity: implications for data harmonization. Scand J Med Sci Sports.

[13] Leis R, Jurado-Castro JM, Llorente-Cantarero FJ (2020). Cluster analysis of physical activity patterns, and relationship with sedentary behavior and healthy lifestyles in prepubertal children: genobox cohort. Nutrients.

[14] Bornstein MH, Bornstein MH (2018). Tanner stages. The SAGE encyclopedia of lifespan human development.

[15] Troiano RP, Berrigan D, Dodd KW (2008). Physical activity in the United States measured by accelerometer. Med Sci Sports Exerc.

[16] Matthews CE, Chen KY, Freedson PS (2008). Amount of time spent in sedentary behaviors in the United States, 2003–2004. Am J Epidemiol.

[17] Choi L, Liu Z, Matthews CE (2011). Validation of accelerometer wear and nonwear time classification algorithm. Med Sci Sports Exerc.

[18] Evenson KR, Catellier DJ, Gill K (2008). Calibration of two objective measures of physical activity for children. J Sports Sci.

[19] Pulsford RM, Cortina-Borja M, Rich C (2011). Actigraph accelerometer-defined boundaries for sedentary behaviour and physical activity intensities in 7 year old children. PLoS ONE.

[20] Freedson P, Pober D, Janz KF (2005). Calibration of accelerometer output for children. Med Sci Sports Exerc.

[21] Puyau MR, Adolph AL, Vohra FA (2002). Validation and calibration of physical activity monitors in children. Obes Res.

[22] Mattocks C, Leary S, Ness A (2007). Calibration of an accelerometer during free-living activities in children. Int J Pediatr Obes.

[23] Peeters G, van Gellecum Y, Ryde G (2013). Is the pain of activity log-books worth the gain in precision when distinguishing wear and non-wear time for tri-axial accelerometers?. J Sci Med Sport.

[24] Vasques C, Mota M, Correia T (2012). Prevalence of overweight/obesity and its association with sedentary behavior in children. Rev Port Cardiol.

[25] Llorente-Cantarero FJ, Aguilar-Gómez FJ, Anguita-Ruiz A (2020). Changes in physical activity patterns from childhood to adolescence: genobox longitudinal study. Int J Environ Res Public Health.

[26] Herman Hansen B, Børtnes I, Hildebrand M (2013). Validity of the ActiGraph GT1M during walking and cycling. J Sports Sci.

[27] Vanhelst J, Béghin L, Duhamel A (2012). Comparison of uniaxial and triaxial accelerometry in the assessment of physical activity among adolescents under free-living conditions: the HELENA study. BMC Med Res Methodol.

[28] Whitaker KM, Gabriel KP, Jacobs DR (2018). Comparison of two generations of ActiGraph accelerometers: the CARDIA Study. Med Sci Sports Exerc.

[29] Jimmy G, Seiler R, Maeder U (2013). Development and validation of energy expenditure prediction models based on GT3X accelerometer data in 5- to 9-year-old children. J Phys Act Health.

[30] Zhu Z, Chen P, Zhuang J (2013). Intensity classification accuracy of accelerometer-measured physical activities in chinese children and youth. Res Q Exerc Sport.

[31] Hildebrand M, Van Hees VT, Hansen BH (2014). Age group comparability of raw accelerometer output from wrist- and hip-worn monitors. Med Sci Sports Exerc.

[32] Hänggi JM, Phillips LRS, Rowlands AV (2013). Validation of the GT3X ActiGraph in children and comparison with the GT1M ActiGraph. J Sci Med Sport.

[33] Loomba-Albrecht LA, Styne DM (2009). Effect of puberty on body composition. Curr Opin Endocrinol Diabetes Obes.

[34] Jurado-Castro JM, Gil-Campos M, Gonzalez-Gonzalez H (2020). Evaluation of physical activity and lifestyle interventions focused on school children with obesity using accelerometry: a systematic review and meta-analysis. Int J Environ Res Public Health.

[35] Robertson W, Stewart-Brown S, Wilcock E (2011). Utility of accelerometers to measure physical activity in children attending an obesity treatment intervention. J Obes.

[36] Dias Moura IR, Barbosa AO, da Silva ICM (2019). Impact of cutoff points on adolescent sedentary behavior measured by accelerometer. Rev Bras Ativ Fís Saúde.

[37] Gába A, Dygrýn J, Mitáš J (2016). Effect of accelerometer cut-off points on the recommended level of physical activity for obesity prevention in children. PLoS ONE.

