# Supplementary material for: Evaluation of Sedentary Behavior and Physical Activity Levels Using Different Accelerometry Protocols in Children from the GENOBOX Study
Source: Sports Med Open. 2021 Nov 24;7:86. doi: 10.1186/s40798-021-00365-z (PMC8613328; doi:10.1186/s40798-021-00365-z)
Supplement: Supplementary file 1 — Additional file 1. Supplementary Figure 1. Bland-Altman plots of sedentary behavior measurement agreement between the Evenson et al. and Freedson et al.; Mattocks et al.: Pulsford et al.; Puyau et al.; Troiano et al. accelerometry protocols. SB; sedentary behavior. Supplementary Figure 2. Bland-Altman plots of light activity measurement agreement between the Evenson et al. and Freedson et al., Mattocks et al., Pulsford et al., Puyau et al., Troiano et al. accelerometry protocols. LPA: light physical activity. Supplementary Figure 3. Bland-Altman plots of moderate physical activity measurement agreement between the Evenson et al. and Freedson et al., Mattocks et al., Pulsford et al., Puyau et al., Troiano et al. accelerometry protocols. MPA: moderate physical activity. Supplementary Figure 4. Bland-Altman plots of vigorous physical activity measurement agreement between the Evenson et al. and Freedson et al., Mattocks et al., Pulsford et al., Puyau et al., Troiano et al. accelerometry protocols. VPA: vigorous physical activity. Supplementary Figure 5. Bland-Altman plots of moderate-vigorous physical activity measurement agreement between the Evenson et al. and Freedson et al., Mattocks et al., Pulsford et al., Puyau et al., Troiano et al. accelerometry protocols. MVPA: moderate-vigorous physical activity. [file 40798_2021_365_MOESM1_ESM.docx]

Sport Medicine – Open

Evaluation of Sedentary Behavior and Physical Activity Levels using Different Accelerometry Protocols in Children from the GENOBOX study

**Francisco Jesus Llorente-Cantarero^1,2†^, Jose Manuel Jurado-Castro^3†^, Rosaura Leis^2,4†^, Rocío Vázquez-Cobela^2,4^_,_ Esther M González-Gil^2,5^_,_ Concepción María Aguilera^2,6,^, Gloria Bueno ^2,5,7*^, Luis A. Moreno^2,5^, Angel Gil^2,6^, Mercedes Gil-Campos^2,3^**

1 Department of Specific Didactics , Faculty of Education, University of Córdoba, Spain.

2 CIBEROBN, (Physiopathology of Obesity and Nutrition) Institute of Health Carlos III (ISCIII); Madrid, Spain.

3 Metabolism and Investigation Unit, Reina Sofia University Hospital. Maimónides Institute of Biomedicine Research of Córdoba (IMIBIC). University of Córdoba, Spain.

4 Pediatric Nutrition Research Group. Institute of Sanitary Research of Santiago de Compostela (IDIS). Unit of Pediatric Gastroenterology, Hepatology and Nutrition. Pediatric Service. University Clinical Hospital of Santiago (CHUS). Santiago de Compostela, Spain.

5 GENUD Research Group, University of Zaragoza, Instituto Agroalimentario de Aragón (IA2), Instituto de Investigación Sanitaria (IIS) Aragón, Zaragoza, Spain.

6 Department of Biochemistry and Molecular Biology II, Institute of Nutrition and Food Technology "José Mataix", Center of Biomedical Research, , University of Granada, Instituto de Investigación Biosanitaria IBS, Granada, Spain.

7 Pediatric Endocrinology Unit, Lozano Blesa University Hospital, University of Zaragoza, Spain.

† Equally contributed

***** Corresponding author:

Gloria Bueno

Unidad de Endocrinología Pediátrica, Hospital Clínico Lozano Blesa,

Universidad de Zaragoza, Spain

mgbuenol@unizar.es;

Tel.: +0034-619223420

|  |
| --- |


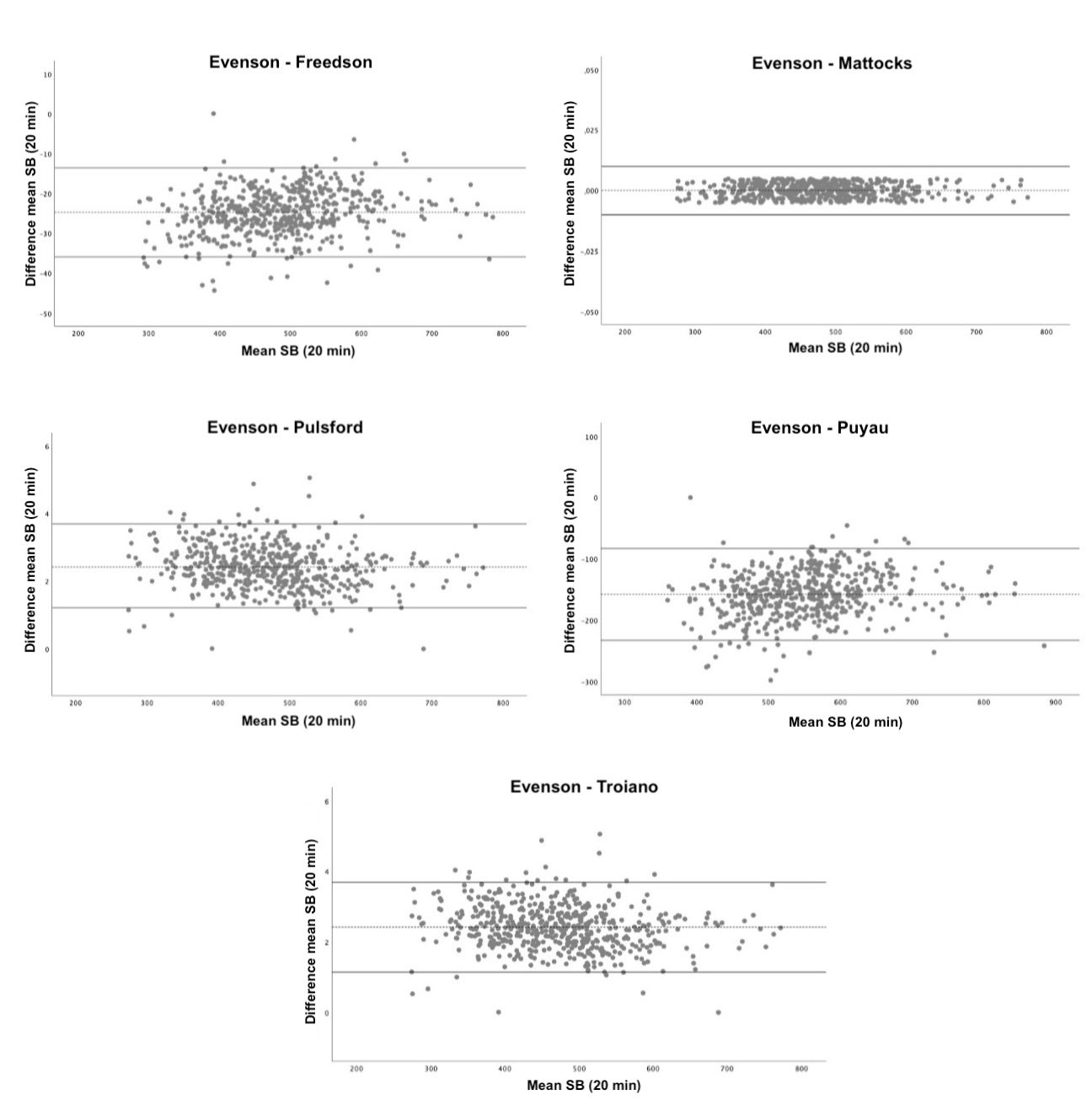


Supplementary Figure 1. Bland-Altman plots of sedentary behavior measurement agreement between the Evenson et al. and Freedson et al.; Mattocks et al.: Pulsford et al.; Puyau et al.; Troiano et al. accelerometry protocols. SB; sedentary behavior.

Note: Data in this figure had the same behavior as that obtained with the 60-min of non-wear-time (results not shown).


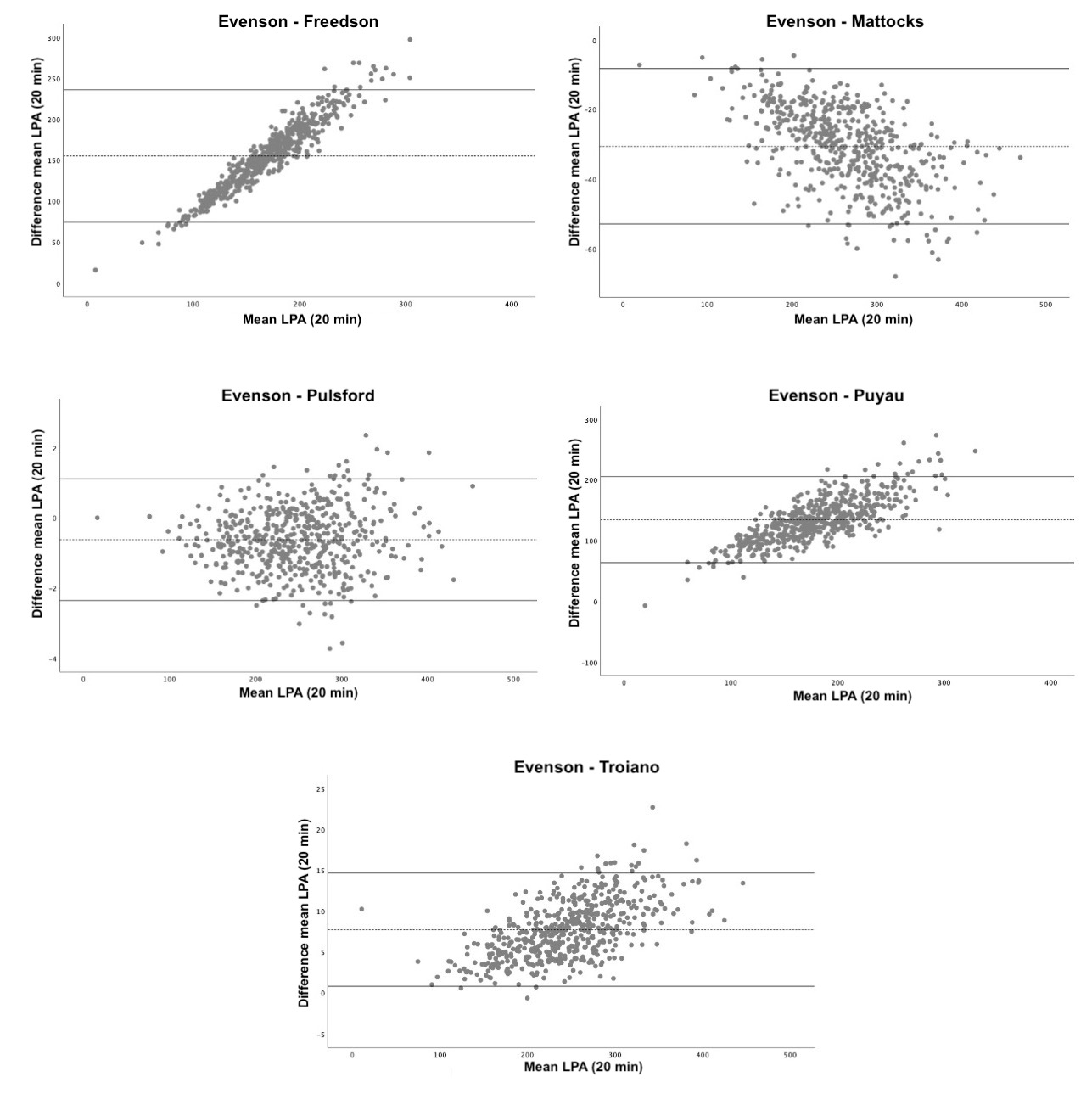


Supplementary Figure 2. Bland-Altman plots of light activity measurement agreement between the Evenson et al. and Freedson et al., Mattocks et al., Pulsford et al., Puyau et al., Troiano et al. accelerometry protocols. LPA: light physical activity.

Note: Data in this figure had the same behavior as that obtained with the 60-min of non-wear-time (results not shown).


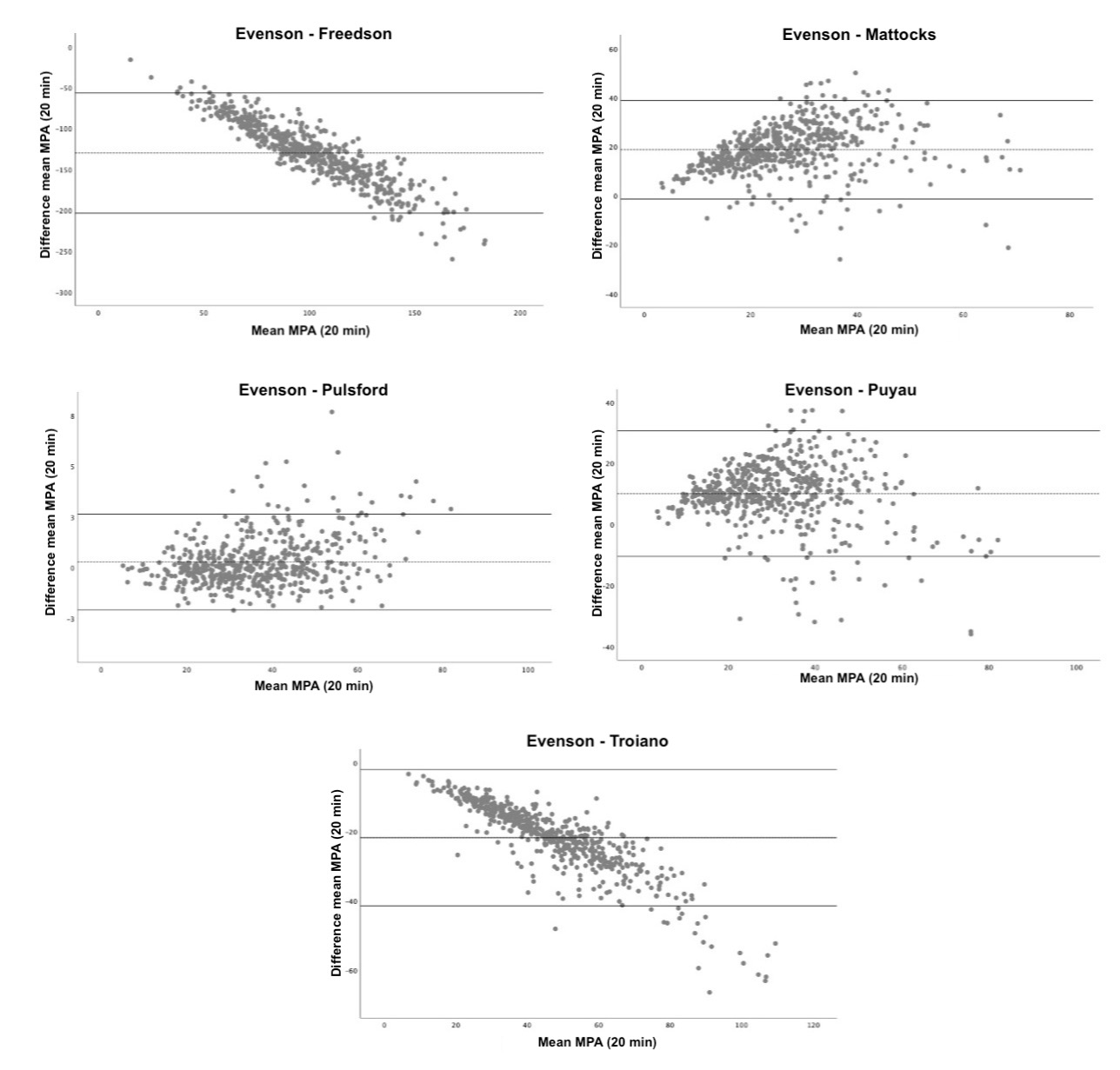


Supplementary Figure 3. Bland-Altman plots of moderate physical activity measurement agreement between the Evenson et al. and Freedson et al., Mattocks et al., Pulsford et al., Puyau et al., Troiano et al. accelerometry protocols. MPA: moderate physical activity

Note: Data in this figure had the same behavior as that obtained with the 60-min of non-wear-time (results not shown).


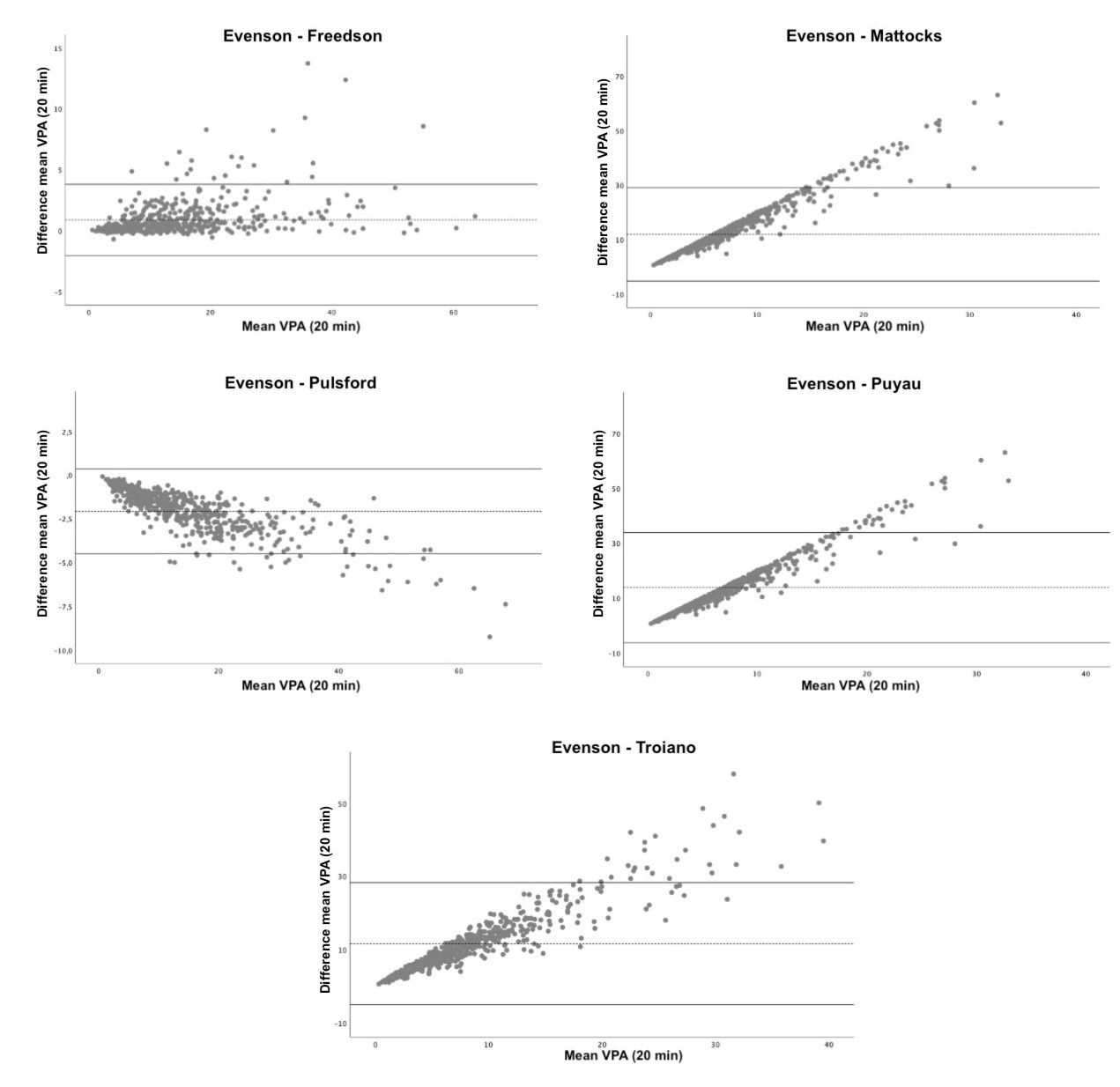


Supplementary Figure 4. Bland-Altman plots of vigorous physical activity measurement agreement between the Evenson et al. and Freedson et al., Mattocks et al., Pulsford et al., Puyau et al., Troiano et al. accelerometry protocols. VPA: vigorous physical activity.

Note: Data in this figure had the same behavior as that obtained with the 60-min of non-wear-time (results not shown).


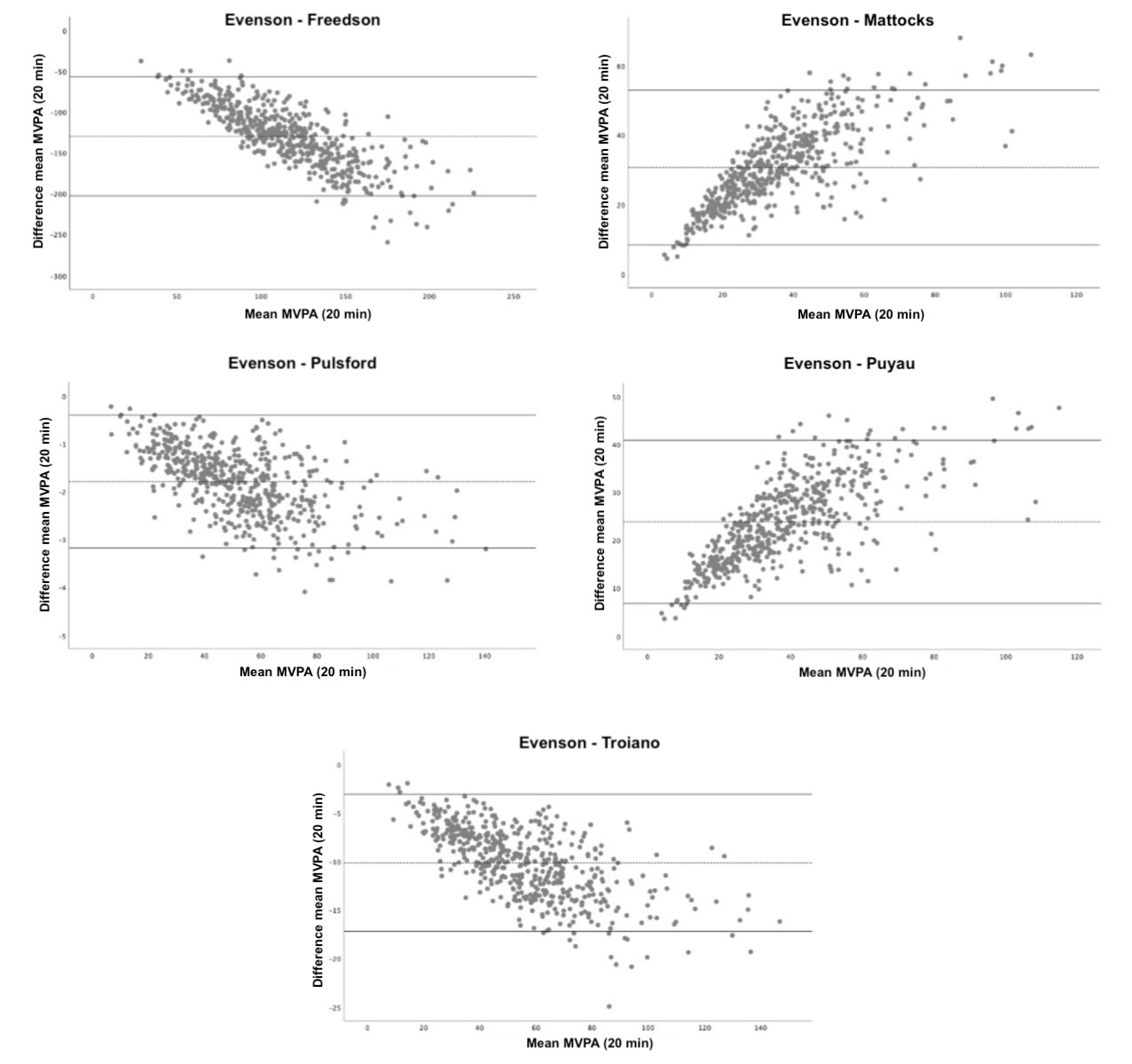


Supplementary Figure 5. Bland-Altman plots of moderate-vigorous physical activity measurement agreement between the Evenson et al. and Freedson et al., Mattocks et al., Pulsford et al., Puyau et al., Troiano et al. accelerometry protocols. MVPA: moderate-vigorous physical activity

Note: Data in this figure had the same behavior as that obtained with the 60-min of non-wear-time (results not shown).
